# Determinants of undernutrition among khat chewing and non-khat chewing male adults in Addis Ababa, Ethiopia: a comparative cross-sectional study

**DOI:** 10.1038/s41598-024-54471-5

**Published:** 2024-02-26

**Authors:** Genene Hailesellasie, Abdu Oumer, Agize Asfaw

**Affiliations:** 1 Department of Public Health, College of Medicine and Health Sciences, Wolkite University, P.O. Box: 07, Gubre, Ethiopia; 2 School of Public Health, College of Medicine and Health Science, Haramaya University, Harar, Ethiopia

**Keywords:** Adults, Comparative study, Ethiopia, Khat chewing, Undernutrition, Health care, Risk factors

## Abstract

Khat chewing is a deep-rooted socio-cultural tradition that affects appetite, gastric emptying, and food intake, ultimately influencing nutritional status. Moreover, there is significant variation in lifestyles and ways of living among khat chewing and non-chewing people. However, there is limited evidence on the disaggregated determinants of undernutrition among khat chewers and non-chewers in Ethiopia. A community-based comparative cross-sectional study was conducted with 253 Khat-chewing and 249 non-chewing male adults in Addis Ababa, Ethiopia. Weight and height were measured under standard procedure and used to calculate the body mass index (BMI < 18.5 kg/m^2^ indicates undernutrition). Variables with p-values below 0.25 in the bi-variable analysis were entered into a multivariable logistic regression model to identify factors associated with undernutrition and to control confounding respectively. An adjusted odds ratio with 95% confidence interval was used to declare the presence and the strength of association between the independent and outcome variable. Statistical significance was declared at a p value of 0.05. In this study, a total of 138 (27.5%: 23.6–31.6%) adult males were undernourished; among them, 78 (32.0%) were khat chewers and 60 (23.9%) were non chewers. Christian religion (AOR = 1.49; 1.02–2.30), vegetable consumption (AOR = 1.69; 95% CI 1.12–2.55) and khat chewing (AOR = 1.60; 1.04–2.45) were independent risk factors for undernutrition. However, none of the above factors showed a statistically significant association among non-khat chewer male adults. In conclusion, undernutrition was a public health concern in male adults in the study area. Frequent consumption of fruits, vegetables and family size of the households were the independent predictors of undernutrition. The practical implication of identifying risk factors of undernutrition among chewers and non-chewers could be helpful in depicting the relevant risk factors by exposure category and helping to further refine intervention packages. In addition, focusing on interventions which can increase the availability and accessibility of fruits and vegetables are important to improve the nutritional status of adult male populations.

## Introduction

Undernutrition is a form of malnutrition characterized by an insufficient intake of energy and/or nutrients to meet an individual's needs to maintain good health^1^. Undernutrition is a major public health problem for people living in underdeveloped regions of the world, particularly Sub-Saharan Africa countries that have not received enough attention^2,3^. In Ethiopia, 33% of adult men and 22% of adult women exhibited undernourishment (Body Mass Index (BMI) < 18.5 kg/m^2^)^4,5^.

However, the risks of undernutrition might vary for different segments of the population. Hence, during each stage of human development, appropriate nutrition is necessary to ensure the optimum overall health of an individual^6^. This could have been aggravated by the consumption of a green plant named: Khat. Khat (Catha edulis) is a green plant that grows throughout the year. It mostly grows at high altitudes of 1500–2500 m, mainly in countries surrounding the Red Sea and along the east coast of Africa, including Ethiopia^7,8^. Khat contains alkaloids of the phenylpropanolamine type, which contains two psychoactive stimulants, cathinone and cathine. In khat chewers, these stimulants are believed to cause excitement, loss of appetite, and euphoria after or during chewing^8,9^. Generally, khat chewing affects many aspects of life with its adverse social, economic, and medical consequences^10^, which may ultimately increase the risks of undernutrition and morbidity.

Undernutrition progressively impairs the function of all systems throughout the body, impairing growth, development and quality of life, ultimately leading to death when weight loss is extreme. Even though, poverty is considered the leading cause of undernutrition, especially in low-income countries, certain other conditions like khat chewing increase the risk of undernutrition. As indicated by previous studies, adults with risky behaviors like khat chewing are the most vulnerable groups of the population for undernutrition due to loss of appetite, inadequate food intake, and a delay in gastric emptying^11–15^. These could further be aggravated by the low affordability of healthy diets, increasing urban food insecurity, rising food price and lower food expenditure among khat chewers^16–18^.

Some previous studies reported the adverse effects of khat-chewing on nutritional status of chewers. However, most of them were not comparative and did not reveal the effect of khat- chewing on chewers and non-chewers. A study conducted in 2011 in Malarden’s university reported that khat chewing leads to loss of appetite which in turn results in malnutrition (8). As revealed by the Ethiopian study conducted in Jimma town, khat chewers had a lower BMI (undernourished)^5^. Another study that compared nutritional status of khat-chewers and non-chewers in Chiro district, eastern Ethiopia, reported that undernutrition was significantly higher among khat chewers (39.0%) than their non-chewer counterparts (22.4%)^16^. However, it is difficult to compare this study finding with our study, since the former was done on lactating women.

Recognizing the problem, the Ethiopian government planned to reduce the prevalence of current khat use in persons aged 15+ years by 20% in 2025^19^. However, the numbers of khat chewing people are increasing from time to time in the country^20–24^. Khat consumption also increases with age and peaks at age 30–34 among both women and men. In addition, khat chewers usually take high-calorie foods as they believe it stays in the stomach for a long time^25^. This coupled with limited physical activity among urban khat chewers makes them at high risk for non-communicable diseases (NCDs). On the other side, the khat consuming population has a poor appetite, limited economic productivity, and poor self-care practices that predispose them to poor intake and increased vulnerability to illnesses^26–28^. As revealed by another previous study, khat chewing could increase the risk of undernutrition by twofolds where 20.5% and 13.5% of khat chewers and non-chewers were undernourished, respectively^29^. A modest effect of khat chewing on stunting among adolescents was also indicated^30^. Furthermore, restrictive dietary behaviors and khat chewing were important predictors of anemia among women^31^.

Despite the fact that khat chewers are one of the most nutritionally vulnerable groups, Ethiopia has limited evidence about the nutritional status of khat chewers as compared to non-chewers. Lack of evidence is more prominent in large cities like Addis Ababa despite consistent increment in khat chewing habits and its negative impact on adult population’s everyday life. Understanding the magnitude of the problem in a local context is essential for relevant interventions. The current study was aimed to fill this gap by generating evidence comparing undernutrition among khat chewer and non-chewer male adults and assessing the prevalence and determinants of undernutrition among them in one of the most urbanized cities in Ethiopia, Addis Ababa.

## Methods

### Study area and design

A community-based cross-sectional study was conducted among the adult population residing in the Kolfe-Keraniyo sub-city of Addis Ababa, Ethiopia. Addis Ababa is the capital and largest city of Ethiopia, located on a plateau surrounded by hills and mountains in the center of the country. It hosts the offices of the African Union and the United Nations Economic Commission for Africa. It has a diverse population, culture, and highland topography at an altitude of 2400 m. The city is comprised of 11 sub-cities with an estimated population of 5,006,000 in 2021.

Khat is the leading source of export business and a major source of income in many parts of the country^32^. It is widely cultivated as a cash crop, mainly in the northeastern, eastern, and southwestern parts of the country^33^. This wide production, along with easy transport access, makes khat more accessible in urban cities.

The city has easy access to Khat from every direction of the country. The diverse ethnicity and cultural background of the population, related to yearly urban migration, makes khat chewing more common, especially among adults. Adult employment is also more prevalent in the city, where many adults could be exposed to substances including khat chewing. The study was conducted from February to March, 2022.

### Eligibility criteria

Adult khat chewers who had used khat at least once in the last month before the study and in the khat chewing shops during the study time and adult non-khat chewing people living in the houses around the khat chewing shops during the study period were participated^34^. All participants in this study were males, as all khat chewers found in the khat chewing shops were males, and subsequently, the comparator group was made to be of male gender only to keep the groups comparable.

### Sample size determination

The sample size was calculated using Epi Info version 7 for the sample size module using the following assumptions: 39.0% and 22.4% prevalence of undernutrition among khat chewers and non-khat chewers from previous similar study, respectively^16^, with 95% certainty, 5% significance level, 80% power of detection, 10% non-response rate, the proportion of khat chewers to non-chewers being 1:1 and considering a design effect of 2, the final calculated sample size for this study was 506.

### Sampling technique

Addis Ababa city is comprised of 11 sub-cities. However, for this study, one randomly selected sub-city was targeted, which could be very representative of the whole city. The selected sub-city (kolfe-keraniyo) in turn consists of 11 smaller administrative levels (kebeles). Using the same sampling technique (SRS), four kebeles were selected, and all khat chewing shops in the selected kebeles were listed. Using the list, we identified 253 khat-chewers from the chewing shops and 249 non-chewers of similar groups engaged in different activities around the shop. In the meantime, stratified random sampling with proportional allocation was employed to select the number of adults for each kebele and study group.

### Data collection procedures

A structured, pretested questionnaire was used for the face-to-face interview. The questionnaire was prepared after reviewing different literatures^2–4,7,9,20,33^. Anthropometric data was collected using standard procedures^35^. Relevant socio-demographic and other data were captured in a standardized manner in local languages. Weight was measured using a portable battery-operated Seca digital scale (Seca, Germany). The weighing scale was adjusted to zero before the adult was asked to stand on it. In addition, the proper functioning of each scale was checked every day by a known 3-kg sand-filled plastic bottle before the fieldwork. During the procedure, the subjects were dressed in light clothes and took off their shoes. The weight was recorded to the nearest 0.1 kg. Height was measured using a portable stadiometer. All participants have been measured against the wall in an upright position, without foot wear, with heels together, their heads positioned in such a way that the posterior part (the occipital prominence) touches the vertical measuring board, and their eyes looking straight ahead (Frankfurt plane), so that the line of sight is perpendicular to the body. The knees are straight, arms at sides, and the heels, buttocks, and shoulder blades touch the vertical surface of the stadiometer. The height was recorded to the nearest 0.1 cm. Both weight and height measurements were taken in duplicate and the average of the two measurements was considered for the final analysis.

The operational definitions given to the most important variables were: ‘*Underweight*’, defined as a condition when the BMI (body mass index) is below 18.5 kg/m^2^, obtained by dividing the weight of an individual in kilograms, to the square of height in meters. It is classified as: ‘underweight’ if BMI < 18.5 kg/m^2^, ‘normal’ if BMI ≥ 18.5 to 24.9 kg/m^2^, ‘overweight’ if BMI ≥ 25.0 to 29.9 kg/m^2^ and obesity if the BMI is 30.0 kg/m^2^ or higher^34^. ‘*Dietary patterns*’, was defined as quantities, proportions, variety, or combination of different foods, drinks, and nutrients in diets and the frequency with which they are habitually consumed in each meal. In this study, a dietary pattern was considered as ‘*poor*’ if an individual consumed two or less meal per day and ‘*good*’ if three or more meals were consumed per day^36^. ‘*Vegetable and fruit consumptions*’, implying a coded responses to vegetable and fruit related questions in the form of servings/week and the level of participant’s consumption of fruits and vegetables. Accordingly, the individual was labeled as ‘*less frequent consumer*’ if he used six or less servings per week and *frequent* if he consumed seven or more servings per week. *Khat chewer*: An individual who chewed khat at least once in the last month before the study and currently actively engaged in khat buying and chewing^37^.

### Data quality control

The questionnaire was prepared in English and then translated into the local language (Amharic). Before using the Amharic version for data collection, a re-translation back to English was made by language experts to check for consistency. Five percent of the sample was pretested in a non-selected sub-city (Gulele) to ensure that respondents were able to understand the questions and to check the wording, logic, and skip order of the questions in a sensible way. A slight modification of the questionnaire was made based on pre-test findings. Two days of demonstration-based training were given for data collectors. Measuring devices were kept at a standard level. Validation and calibration of the instrument after each measurement and after moving the instrument from one place to another were performed. Data collectors were recruited based on their qualifications, fluency in local language for effective communication and prior experience in data collection. All the procedures were checked for consistency and accuracy during supervision periods.

### Data processing and analysis

After data collection, each questionnaire was checked manually for completeness, missed values, and unlikely responses, then coded, and entered using Epi-data version 3.1 for data exploration and cleaning. The cleaned data was exported to SPSS version 23.0 for statistical analysis. Descriptive statistics were computed to determine frequencies and summary statistics (mean, standard deviation, and percentage) to describe the study population. Data were presented using tables, graphs, and figures. Bi-variable logistic regression analysis was used to show the association between dependent and predictor variables. Variables that were found to be statistically significant at p-value < 0.25 during bi-variable analysis were considered for further analysis in multivariable logistic regression model. Adjusted odds ratios with their 95% CI was computed and variables having p-value less than or equal to 0.05 in the multivariable logistic regression model were considered statistically significant and independent predictors of the outcome variable. Hosmer–Lemeshow’s goodness-of-fit statistics^38,39^ were conducted to determine whether the model adequately describes the data (p-value of 0.53 indicating a fit model).

### Ethical approval

The ethical approval was obtained from the Institutional Review Board of Wolkite University, College of Medicine and Health Sciences. Then, the detailed purpose of the study was explained to all study participants, and written informed consent was obtained from each participant. All information collected from the respondents was kept confidential by the investigator. The participants had the right to withdraw from participation whenever they wanted to. A work permission letter was obtained from the office of the local health bureau and all methods and procedures were conducted in accordance with the approved ethical standard and, with respect to studies involving human subjects, in accordance with the Helsinki Declaration.

## Results

### Socio-demographic characteristics

A total of 502 individuals participated in this study (253 khat chewers and 249 non-chewers). Among the non-khat chewer participants, four individuals refused to answer the questions, giving a response rate of 99.2%. The mean age ± Standard deviation of participants was 37.5 ± 11.29 years. The leading khat chewers were in the age group 15–24 years, constituting 269 (67.8%), followed by the age group 25–64 years, constituting 108 (27.2%). These two, together comprising a total of 95.0% of khat chewers, were in the active and productive age groups. More than half of khat chewers, 226 (56.9%) and 241 (60.7%) were married and college graduates, respectively). About two-thirds of the respondent’s average monthly income lies between 2500 and 5000 Ethiopian birr per month for both groups (US$43–92). Regarding family size, half of the respondents in the khat-chewer group had small families size (1–3). In addition, there was no statistically significant difference in the baseline characteristics of study participants among khat chewers and non-khat chewers (Table 1).

**Table 1:** Socio-demographic characteristics of adult male Khat chewers and non-Khat chewers in Addis Ababa, Ethiopia, 2022.

Variables	Category	Khat chewers	Non-chewers	X^2^ (df)	P-value
		Number (%)	Number (%)		
Age groups (years)	15–24	269 (67.8)	69 (65.7)	0.009 (2)	0.995
	25–64	108 (27.2)	28 (26.7)		
	> 64	20 (5.0)	8 (7.6)		
Religion	Orthodox	49 (46.7)	217 (54.7)	0.001 (1)	0.991
	Muslim	56 (53.3)	180 (45.3)		
Educational level	College and above	241 (60.7)	70 (66.7)	0.018 (1)	0.892
	Below college level	156 (39.3)	35 (33.3)		
Employment type	Employed	41 (10.3)	10 (9.7)	0.043 (1)	0.835
	Not employed	356 (89.7)	95 (90.5)		
Average monthly income	< 2500	6 (1.5)	1 (1.0)	2.33 (4)	0.675
	2500–4999	256 (64.5)	68 (64.8)		
	5000–9999	117 (29.5)	30 (28.6)		
	> 10,000	18 (4.6)	6 (5.8)		
Marital status	Married	226 (56.9)	57 (54.3)	0.006 (2)	0.997
	Unmarried	138 (34.8)	35 (33.3)		
	Separated	33 (8.3)	13 (12.4)		
Family size	1–3	199 (50.1)	47 (44.8)	0.006 (2)	0.997
	4–6	137 (34.5)	45 (42.9)		
	> 6	61 (15.4)	13 (12.4)		

### Diet-related characteristics of khat chewers and non-chewers

Among the factors affecting nutritional status, khat chewing showed a significant influence on loss of appetite 89 (35.2%, p 0.001), with a lower number of meals per day in more than half (54.9%) of chewers (p = 0.007). This in turn affected the dietary consumption pattern of chewers (139, 54.9%, p 0.001). One hundred fifty-seven (62.1%) of Khat chewers reported to consume fewer fluids (5 cups per day) as compared to the non-chewers (48.2%). Furthermore, khat chewers showed a lesser consumption of vegetables, fruits, and animal products when compared to non-chewers (43.5% vs. 57.8%; 36.8 vs. 53.4%; and 25.7% vs. 41.0%, respectively) (Table 2).

**Table 2:** Diet-related factors and substance use between khat chewer and non-chewer in Addis Ababa, Ethiopia, 2022.

Variables	Category	Khat chewers	Non-chewers	P-value
		Number (%)	Number (%)	
Dietary pattern	Poor	139 (54.9)	96 (38.6)	< 0.001
	Good	114 (45.1)	153 (61.4)	
Meals per day	< 3	139 (54.9)	108 (43.4)	0.007
	≥ 3	114 (45.1)	141 (56.6)	
Loss of appetite	Yes	89 (35.2)	42 (16.9)	< 0.001
	No	164 (64.8)	207 (83.1)	
Fluid intake (cups/day)	< 5	157 (62.1)	120 (48.2)	0.007
	≥ 5	96 (37.9)	129 (51.8)	
Consume animal products	Yes	65 (25.7)	102 (41.0)	< 0.001
	No	188 (74.3)	147 (59.0)	
Consume vegetables	Yes	110 (43.5)	144 (57.8)	< 0.001
	No	143 (56.5)	105 (42.2)	
Consume fruits	Yes	93 (36.8)	133 (53.4)	0.002
	No	160 (63.2)	116 (46.6)	
Drink alcohol	Yes	144 (56.9)	109 (43.8)	0.019
	No	109 (43.1)	140 (56.2)	
Smoke cigarettes	Yes	56 (22.1)	25 (10.0)	< 0.001
	No	197 (77.9)	224 (90.0)	

### Prevalence of undernutrition among khat chewers and non-chewers

**Figure 1:**
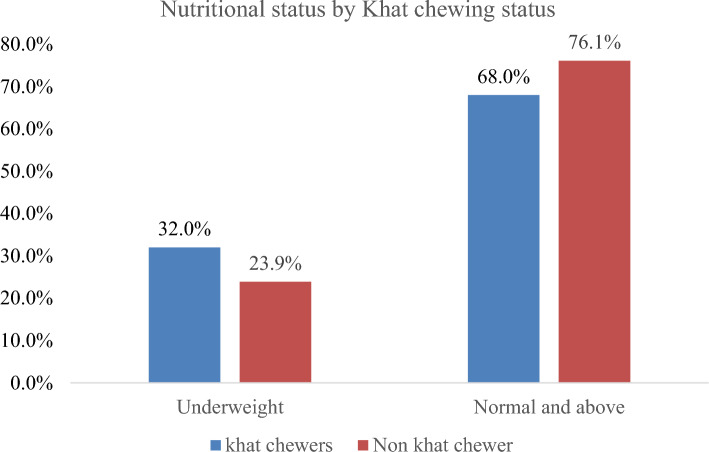
Prevalence of undernutrition by the khat chewing status of adult male in Addis Ababa, Ethiopia, 2022.

A total of 138 (27.5%; 95% CI 23.6–31.6%) adult males were undernourished with a BMI below 18.5 kg/m^2^. When disaggregated by chewing status, the prevalence of undernutrition (underweight) was higher among khat chewers 78 (32.0%; 95% CI 25.2–36.9) than their non-chewer counterparts 60 (23.9%; 95% CI 18.9–28.9%), but the difference is not statistically significant (Fig. 1). In this study, overweight was negligible as only three individuals (0.6%) were overweight and only one of them was a khat chewer.

### Factors associated with undernutrition

In the regression analysis model, we employed three separate models. While the first was to explore the determinants of undernutrition (khat chewing included) among the general population, the latter two involve separate models among khat chewers and non-chewers. We explored the factors associated with undernutrition after checking for model fitness using Hosmer and Lemeshow’s test (p = 0.622).

First, bi-variable logistic regression analysis was conducted and all variables with a p-value ≤ 0.25 in the bi-variable analysis were fitted into the multivariable logistic regression analysis. Accordingly, religion, vegetable consumption per week, and khat chewing were found to be significant predictors of undernutrition at p-values ≤ 0.05. Being Christian in religion (AOR = 1.49; 95% CI 1.02–2.30) and having frequent vegetable intakes per week (AOR = 1.69; 95% CI 1.12–2.55) were associated with 1.49 times and 1.69 times increased risk of undernutrition, respectively. More importantly, khat chewing was significantly associated with a 60% increased risk of being undernourished (AOR = 1.60; 95% CI 1.04–2.45). This indicated that khat chewing is an important determining factor for undernutrition among adult males **(**Table 3).

**Table 3:** Bi-variable and multivariable logistic regression output for factors associated with undernutrition among adult males in Addis Ababa, Ethiopia, 2022.

Factors	Options	Nutritional status		COR 95% CI	
		Undernourished	Normal		AOR 95% CI
Age	15–24	95	243	1.80 (0.66–4.87)	^¶^
	25–64	38	98	1.78 (0.63–5.03)	
	> 64	5	23	1	
Educational status	Below college	59	132	1.31 (0.88–1.96)	^¶^
	College graduate	79	232	1	
Marital status	Unmarried	48	125	1.07 (0.70–1.63)	^¶^
	Separated	15	31	1.34 (0.67–2.62)	^¶^
	Married	75	208	1	
Religion	Christians	83	183	1.49 (1.00–2.22)	1.49 (1.02–2.30)*
	Muslim	55	181	1	1
Alcohol	Yes	68	181	0.98 (0.66–1.45)	^¶^
	No	70	183	1	
Smoking	Yes	121	300	1.52 (0.85–2.70)	^¶^
	No	17	64	1	
vegetable intake per week	Frequent	79	169	1.55 (1.04–2.29)	1.69 (1.12–2.55)*
	Less frequent	59	195	1	1
Fruits consumed per week	Frequent	78	198	1.09 (0.73–1.62)	^¶^
	Less frequent	60	166	1	
Khat chewing	Yes	78	171	1.47 (0.99–2.18)	1.60 (1.04–2.45)*
	No	60	193	1	1
Loss of appetite	Yes	38	93	1.11 (0.71–1.72)	^¶^
	No	100	271	1	

^¶^No significant association at P < 0.05.

*Significant association at P < 0.05.

### Factors associated with undernutrition among khat chewers and non-chewers (comparison)

As presented above, khat chewing was a main determinant factor for undernutrition in this study (Table 3). In this section, we tried to compare factors other than khat chewing in relation to undernutrition in khat chewers and their non-chewer counterparts.

Among khat chewers, religion, family size, and vegetable and fruit consumption were important and independent predictors of undernutrition. Christian religion followers (AOR = 1.90; 1.04–3.44), less frequent vegetable (AOR = 2.72; 95% CI 1.51–4.88), and fruit consumers (AOR = 1.87; 95% CI 1.05–3.32) per week had a 90%, 72%, and 87% higher risk of being undernourished, respectively, in khat chewers. Living in a medium-sized family (AOR = 0.50; 95% CI 0.26–0.96) showed a 50% protective tendency from undernutrition when compared with the extended family in chewers. However, none of the above factors showed a statistically significant association among non-khat chewers male adults (Table 4).

**Table 4:** Multivariable logistic regression output for factors associated with undernutrition among khat chewers and non-chewers in Addis Ababa, Ethiopia, 2022.

Factors	Options	Nutritional status of khat chewers			Nutritional status of non-khat chewers		
		Undernutrition	Normal	AOR	Undernutrition	Normal	AOR
Religion	Christians	47	85	1.90 (1.04–3.44)*	36	98	1.18 (0.93–3.13)
	Muslim	31	86	1	24	95	1
Family size	1–3	35	92	0.50 (0.26–0.96)*	65	75	1.50 (0.80–1.74)
	≥ 4	43	79	1	54	59	1
Fluid intakes	≥ 8cups	42	87	1.28 (0.72–2.27)	24	72	1.30 (0.70–2.41)
	< 8 cups	36	84	1	36	121	1
Consume animal products	Yes	51	96	1.75 (0.96–3.20)	52	97	1.38 (0.74–3.47)
	No	27	75	1	29	75	1
Vegetable intake per week	Frequent	45	60	2.72 (1.51–4.88)*	42	87	0.73 (0.71–2.37)
	Less freq.	33	111	1	40	84	1
Fruits intake per week	Frequent	44	72	1.87 (1.05–3.32)*	26	67	1.52 (0.83–2.78)
	Less freq.	34	99	1	34	126	1

Statistically significant at p-value below 0.05 (*), *Freq.* frequent.

## Discussion

The findings from this study revealed that the overall prevalence of undernutrition among adult males was 27.5%. This finding was in line with the studies conducted in Gulalle sub-city, Addis Ababa^29^, Chiro district, eastern Ethiopia^16^, and Jimma town, southwest Ethiopia^5^. The similarity of the findings could be due to the similarity in the study area and participant population. When disaggregated by khat chewing status, the prevalence of undernutrition was higher among khat chewers (32.0%) than their non-chewer counterparts (23.9%), but such difference was not statistically significant in current study. Similar findings were reported by different researches conducted in Brazil, Taiwan, and among slum dwellers in Guwahati. As revealed by all of these researches, chewing khat was not significantly associated with BMI^9,13,15^ which was in agreement with our study findings.

Khat chewers are usually prone to concurrent consumption of large volumes of sugary drinks and sweets to counteract the bitter taste of khat. Most ultra-processed foods and sugar-sweetened beverages are categorized as unhealthy foods. Frequent consumption of these foods has been associated with undernutrition and vitamin deficiencies by providing excess calories while lacking important micronutrients. This coupled with reduced appetite and poor nutrition further exacerbates the nutritional disturbances^29^.

Concerning factors associated with undernutrition, the vegetable and fruit intake of khat chewer adults was found to be associated with undernutrition. Those adults whose vegetable and fruit intake were below the optimum were more likely to be undernourished as compared to those who were taking optimum fruits and vegetables. This was comparable with the studies conducted in Addis Ababa and eastern Ethiopia^16,29^. The reason behind this might be that adults with low intakes of vegetables and fruit may not get adequate nutrients in their diets that meet the increased nutrient requirements, which in turn results in undernutrition due to depletion.

In the current study, we found that the odds of undernutrition were higher among Christian khat chewers (AOR = 1.90; 95% CI 1.04–3.44) as compared to their Muslim counterparts. Contrary to our finding, previous studies reported a higher prevalence of undernutrition among Muslim khat chewers^20–22^ compared to Orthodox Christians. Another national study estimated that 23.6% of adults consume khat, and the odds of khat chewing increased by 18-fold among Muslim followers^19^. Hence, these might indicate the role of extraneous factors like season (since this study was conducted from February to March which is fasting time for Easter in orthodox Christians), limiting actual food intakes, and repeated fasting and animal-source food restrictions. In addition, the smaller number of Christian khat chewers might be more addicted, with limited adaptation affecting their actual food intakes^28^ and predispose them to undernutrition^14^.

Another factor that partly determines undernutrition among khat chewers is family size. Adult khat chewers who had extended family (≥ 4 members) were more likely to become undernourished as compared to those who had fewer family numbers (1–3). A comparable study from Nekemte, Ethiopia, revealed that participants who had a larger family were five times more likely to be underweight^11^. Another study conducted in Arba Minch Zuria district, southern Ethiopia, showed similar findings. According to the study, lactating mothers who had a family size of 4–6 members were 2.3 times more likely to be undernourished compared to those who had a family size of 1–3 members^12^.

The findings of this study have wide practical implications. Reducing the khat chewing habit in adults could have an important effect on their health and, in turn, on the economic development of the nation. It is important to have such information on khat chewing practices to identify predisposing factors associated with it and draw due attention to alleviate the negative impacts of khat chewing on the individual, family, and society as a whole. Finally, the study was not free of limitations. In this study, generalization of the findings was limited as all the participants were adult males recruited from an urban setting. Another limitation could be under-reporting, as some of the respondents might hide their actual feeding practices or khat-chewing habits. Since the data collection was based on self-report, it may be subjected to recall bias. However, due attention was given to the entire procedure, and since the respondents were probed, its effect might not be a threat to the findings of the study.

## Conclusion

Overall, we tried to quantify the burden of undernutrition and modifiable risk factors for the development of undernutrition among two comparative groups (khat chewers and non-chewers). Hence, undernutrition was a public health concern among male adults in the study area. Khat chewing was found to be a significant predictor of undernutrition in addition to dietary consumption of fruits, vegetables and family size of the households. Moreover, identifying risk factors of undernutrition among chewers and non-chewers could be helpful in depicting the relevant risk factors by exposure category and helping to further refine intervention packages. Based on the finding, the following recommendations can be drawn for government, health professionals and future researchers. Government should give due emphasis on the harmful effects of khat chewing beyond its potential source of export income. Health professionals working at community and health facility level should counsel adult khat chewers about the negative effect of khat on health and to reduce the habit of chewing khat. We also suggest future researchers in the same topic to consider feminine gender in rural community, since this study addressed only male adult with urban setting.

## Data Availability

All the data generated in this study are within the submitted manuscript. Further datasets can be shared by the corresponding author upon reasonable request.

## Abbreviations

AOR: Adjusted odds ratio
BMI: Body mass index
CI: Confidence interval
COR: Crude odds ratio
CSA: Central Statistical Agency
ETB: Ethiopian Birr
FAO: Food and Agricultural Organization
IFAD: International Fund for Agricultural Development
NCDs: Non-communicable diseases
UNICEF: United Nations Children’s Education Fund
US$: United States dollar
WFP: World Food Program

## References

[1] Julla BW, Haile A, Ayana G (2018). Chronic energy deficiency and associated factors among lactating mothers (15–49 years old) in Offa woreda, Wolayita zone, SNNPRs, Ethiopia. World Sci. Res.

[2] FAO, IFAD, UNICEF, WFP and WHO. The State of Food Security and Nutrition in the World. Building resilience for peace and food security. https://www.fao.org/3/I7695e/I7695e.pdf (FAO, 2017).

[3] World health statistics overview. Monitoring health for the SDGs, sustainable development goals. (WHO/DAD/2019.1). Licence: CC BY-NC-SA 3.0 IGO. https://apps.who.int/iris/bitstream/handle/10665/324835/9789241565707-eng.pdf?ua=1 (World Health Organization, 2019).

[4] Central Statistical Agency (CSA) [Ethiopia] and ICF. Ethiopia Demographic and Health Survey 2016. https://dhsprogram.com/pubs/pdf/FR328/FR328.pdf (CSA and ICF, 2016).

[5] Girma T, Mossie A, Getu Y (2015). Association between body composition and khat chewing in Ethiopian adults. BMC Res. Notes..

[6] Smith ML (2020). A brief intervention for malnutrition among older adults: Stepping up your nutrition. Int. J. Environ. Res. Public Health.

[7] Abebe M, Kindie S, Adane K (2015). Adverse health effects of khat: A review. Fam. Med. Med. Sci. Res..

[8] Yusuf, B. The health risks of khat and influences it has on integration issues. Mälardalen University, https://www.semanticscholar.org/paper/The-health-risks-of-Khat&Influences-it-has-onFarah/53d0b39bccb2b3ff35ac567f1b389b6e14f54662 (2011).

[9] Escobar M, Scherer JN, Soares CM (2018). Active Brazilian crack cocaine users: Nutritional, anthropometric, and drug use profiles. Braz. J. Psychiatry..

[10] Wondemagegn AT, Cheme MC, Kibret KT (2017). Perceived psychological, economic, and social impact of khat chewing among adolescents and adults in Nekemte Town, West Ethiopia. Biomed. Res. Int..

[11] Hundera TD, Gemede HF, Wirtu D, Kenie DN (2015). Nutritional status and associated factors among lactating mothers in Nekemte referral hospital and health centers, Ethiopia. Int. J. Nutr. Food Sci..

[12] Kejela G, Gebremeskel F, Hassen H (2020). Under nutrition and associated factors among lactating mothers in Southern Ethiopia: Institution based cross-sectional study. J. Womens Health Saf. Res..

[13] Liu T, Yen J, Ko C, Huang M, Wang P, Yeh Y (2010). Associations between substance use and body mass index: Moderating effects of sociodemographic characteristics. Kaohsiung J. Med. Sci..

[14] Tessema ZT, Zeleke TA (2020). Prevalence and predictors of alcohol use among adult males in Ethiopia: Multilevel analysis of Ethiopian Demographic and Health Survey 2016. Trop. Med. Health.

[15] Tanusri B, Saikia AM, Baruah R (2016). Nutritional status and its relationship with substance use behavior among adolescents slum dwellers of Guwahati. Int. J. Health Res. Medico Legal Pract..

[16] Minas S, Ayele BH, Sisay M, Fage SG (2022). Undernutrition among khat-chewer and non-chewer lactating women in Chiro district, eastern Ethiopia: Comparative cross-sectional study. SAGE Open Med..

[17] Gudata ZG (2020). Khat culture and economic wellbeing: Comparison of a chewer and non-chewer families in Harar city. Cogent .Soc. Sci..

[18] Bachewe F, Hirvonen K, Minten B, Yimer F (2017). The Rising Costs of Nutritious Foods in Ethiopia.

[19] Ethiopian Public Health Institute (EPHI). Ethiopia national strategic plan for the prevention and control of major non-communicable diseases. http://dataverse.nipn.ephi.gov.et/handle/123456789/1425 (2020).

[20] Akalu, T.Y., Baraki, A.G., Wolde, H.F. *et al.* Factors affecting current khat chewing among male adults 15–59 years in Ethiopia, 2016: A multi-level analysis from Ethiopian Demographic Health Survey. *BMC Psychiatry* **20**, 21. 10.1186/s12888-020-2434-7 (2020). 10.1186/s12888-020-2434-7 PMC6961402 31937273

[21] Rather RA, Berhanu S, Abaynah L, Sultan M (2021). Prevalence of khat (*Catha edulis*) chewing and its determinants: A respondent-driven survey from Hossana, Ethiopia. Subst. Abuse Rehabil..

[22] Haile D, Lakew Y (2015). Khat chewing practice and associated factors among adults in Ethiopia: Further analysis using the 2011 Demographic and Health Survey. PLoS ONE.

[23] Reda AA, Moges A, Biadgilign S, Wondmagegn BY (2012). Prevalence and determinants of khat (*Catha edulis*) chewing among high school students in eastern Ethiopia: A cross-sectional study. PLoS ONE.

[24] Gezahegn, E., Edris, M. & Dachew, B. A. Prevalence and factors associated with undernutrition among adults with major depressive disorder in Northwest Ethiopia. *Psychiatry J.* **2016**, 7. 10.1155/2016/7034582 (2016). 10.1155/2016/7034582 PMC5136665 27990420

[25] Alsayegh AA, Chandika RM, Tubaigi AA (2021). Dietary patterns among Khat chewing students at Jazan University, KSA. Indian J. Nutr..

[26] Balint EE, Falkay G, Balint GA (2009). Khat—a controversial plant. Wiener Klinische Wochenschrift.

[27] Engidawork E (2017). Pharmacological and toxicological effects of *Catha edulis* F. (Khat). Phytother. Res..

[28] Boylston T (2013). Food, life, and material religion in Ethiopian Orthodox Christianity. Companion Anthropol. Relig..

[29] Legesse TG, Bedane DG (2016). Prevalence of under nutrition and associated factors among khat chewers in khat chewing shops at Gulalle Sub City, Addis Ababa, Ethiopia. J. Pharm. Nutr. Sci..

[30] Teji Roba K, Brewis A, Manning M, Hassen JY (2023). Parental khat use and early childhood growth status in Eastern Ethiopia. Nutr. Health..

[31] Kedir H, Berhane Y, Worku A (2013). Khat chewing and restrictive dietary behaviors are associated with anemia among pregnant women in high prevalence rural communities in eastern Ethiopia. PLoS ONE.

[32] Cochrane L, O’Regan D (2016). Legal harvest and illegal trade: Trends, challenges, and options in khat production in Ethiopia. Int. J. Drug Policy.

[33] Gebissa E (2010). Khat in the Horn of Africa: Historical perspectives and current trends. J. Ethno Pharmacol..

[34] Weir, C. B. & Jan, A. BMI classification percentile and cut off points. In *StatPearls* (StatPearls Publishing, 2024). 31082114

[35] Kumar A, Kulchar RJ, Khadka N (2023). Maternal–child consumption of ultra-processed foods and sugar-sweetened beverages in informal settlements in Mumbai, India. J. Health Popul. Nutr..

[36] Leech RM, Worsley A, Timperio A, McNaughton SA (2015). Understanding meal patterns: Definitions, methodology and impact on nutrient intake and diet quality. Nutr. Res. Rev..

[37] Adane T, Worku W, Azanaw J, Yohannes L (2021). Khat chewing practice and associated factors among medical students in Gondar Town, Ethiopia, 2019. Subst. Abuse Res. Treat..

[38] Fagerland MW, Hosmer DW (2012). A generalized Hosmer-Lemeshow goodness-of-fit test for multinomial logistic regression models. Stata J..

[39] Nattino G, Pennell ML, Lemeshow S (2020). Assessing the goodness of fit of logistic regression models in large samples: A modification of the Hosmer-Lemeshow test. Biometrics.

